# RNA-Seq of Plant-Parasitic Nematode *Meloidogyne incognita* at Various Stages of Its Development

**DOI:** 10.3389/fgene.2017.00190

**Published:** 2017-11-27

**Authors:** Inchan Choi, Parthiban Subramanian, Donghwan Shim, Byung-Ju Oh, Bum-Soo Hahn

**Affiliations:** ^1^Department of Agricultural Biotechnology, National Institute of Agricultural Sciences, Rural Development Administration, Jeonju, South Korea; ^2^Department of Forest Genetic Resources, National Institute of Forest Science, Suwon, South Korea

**Keywords:** plant parasite, root-knot nematode, developmental stages, RNA-Seq, transcriptome

## Introduction

*Meloidogyne incognita* is a plant-parasitic nematode which attacks a diverse variety of plants (Mitkowski and Abawi, 2003). The life cycle of this nematode is comprised of five stages namely eggs, J2, J3, and J4 juveniles and adults. Female adults are important in the life cycle as they produce eggs which hatch into infective J2 that travels and attaches to plant roots for continuation of its infective cycle. Among the stages of its life cycle, the J3, J4, and female are sedentary and occur after the motile J2 infects a plant root. It is an obligate plant-parasite and possesses a wide host range of over 100 plants including cotton, tobacco, legumes, vegetable crops, spices, and coffee. It also is one of the most widely spread plant pest and has been reported from Asia, Africa, North, Central and South America, the Caribbean, Europe and Oceania (CABI Data Report; CABI, 2017).

Hitherto employed methods have made use of nematicides that can lead to environmental pollution and their use has been banned in developed countries (Collange et al., 2011). Molecular studies on the nematode can help us understand the development and physiology of nematode which can be used to develop control strategies in the future. There have been earlier reports on the transcriptome from egg and J2 juveniles of *M. incognita* and from infected plant tissues (McCarter et al., 2003; Neveu et al., 2003; Dubreuil et al., 2007; Schaff et al., 2007). The present report was aimed at expanding the currently available information on transcriptome of *M*. *incognita*. We aimed to study RNA-Seq data independently for the endophytic J3, J4, and female stages, as well as exophytic egg and J2 stages of the nematode.

## Value of the data

- *M. incognita* is a notorious plant parasite causing huge economic loss in agricultural crops.
- Five morphologically distinct developmental stages of the nematode were selected to collect samples and respective transcriptomes were sequenced.
- The present data can be used to compare and confirm previously reported partial transcriptome of *M. incognita* as well be a useful resource to study the stage-wise changes occurring in the nematode.

## Data

We used RNA-Seq to compare the transcriptomes of five stages namely eggs, J2, J3, J4, and female of the root-knot nematode. Samples were collected, RNA isolated using LiCl_2_ method and RNA sequencing was carried out using HiSeq2500 platform from Illumina Inc. RNA integrity number (RIN) for the samples were >7 (8.5, 9, 9.4, 9.4, 7.8 for egg, J2, J3, J4, and female stages respectively). stage consisted of the highest number of clean reads at 56,902,902 and J2, J3, J4, and female stages consisted of 50,762,456; 40,968,532; 47,309,223, and 51,730,234 clean reads, respectively (Table 1). Three runs were performed for each stage and raw sequences were submitted to Sequence Read Archive (SRA). Contigs were obtained by assembly using TRINITY software and data deposited to the NABIC sever (http://nabic.rda.go.kr/) maintained by Rural Development Administration, Korea (Seol et al., 2016). We were able to identify 48,024 contigs in egg stage followed by 61,315 in J2 stage, 60,697 in J3; 48,398 contigs in J4 and 41,489 contigs in female stage. The accession numbers and other related statistics are given in Table 1. The raw data was submitted to NCBI SRA portal and the accession number for egg stage are SRR5684407, SRR5684403, and SRR5684417; J2 stage—SRR5684416, SRR5684412, and SRR5684414; J3 stage—SRR5684413, SRR5684415, and SRR5684404; J4 stage—SRR5684408, SRR5684406, and SRR5684405; female stage—SRR5684410, SRR5684409, and SRR5684411.

**Table 1:** Summary of sequencing, process, assembly, and submission of *M. incognita* transcriptome at its developmental stages.

**Attributes**	**Egg**	**J2**	**J3**	**J4**	**Female**	**Combined**
**PRE-ASSEMBLY**						
Raw reads	70,310,943	61,150,827	51,342,127	58,016,948	64,618,511	
Clean reads	56,902,902	50,762,456	40,968,532	47,309,223	51,730,234	
NCBI bioproject ID	PRJNA390559	PRJNA390559	PRJNA390559	PRJNA390559	PRJNA390559	
NCBI biosample IDs	SAMN07174878 SAMN07174879 SAMN07174880	SAMN07174881 SAMN07174882 SAMN07174883	SAMN07174884 SAMN07174885 SAMN07174886	SAMN07174965 SAMN07174966 SAMN07174967	SAMN07174968 SAMN07174969 SAMN07174970	
Number of cleaned reads mapped to genome	27,285,236	25,208,092	19,962,672	24,502,219	26,329,321	
Total bases of cleaned reads mapped to genome	5,510,011,716	5,090,859,842	4,031,018,384	4,276,893,153	5,317,234,914	
**POST-ASSEMBLY**						
NABIC accession number	NN-3882-000001	NN-3881-000001	NN-3883-000001	NN-3884-000001	NN-3885-000001	
Number of contigs	48,024	61,315	60,697	48,398	41,489	113,421
N50 (bp)	900	973	780	1,142	1,023	1,338
Number of contigs mapped to genome	37,330	45,981	41,515	40,190	32,531	197,514

## Materials and methods

### Nematode propagation and collection

Root-knot nematode *M. incognita* was maintained in tomato plants (*Solanum lycopersicum* var. Rutgers) under greenhouse conditions (25°C, 16/8 h day/night period). For collection of the nematode at its developmental stages, a new batch of tomato plants were infected. Eggs used for infection were collected by harvesting and treatment of the infected roots with 10% NaClO for 5 min followed by continuous washing over a series of filters 100, 25 μM mesh filter to trap the eggs. Around 1,000 eggs were used to infect each plant and the newly infected plants were continuously monitored to confirm the stage of nematodes in the roots.

Sample collection was carried out in the following order. Around 2 and 6 weeks after infection presence of J3, J4, and female stage nematodes was identified by manual inspection of root-knots under a stereo microscope (Discovery v12, Zeiss Germany). Eggs samples were collected from egg masses and purified by sucrose gradient centrifugation (35% sucrose, 1,500 rpm and room temperature). J2 samples were obtained by hatching a portion of the collected eggs. Eggs of the nematodes were suspended in sterile double distilled water at 25°C for five days and hatched J2 were filtered using 5–7 KimWipes® onto clean Petri dish placed on a lab table. Nematodes at J3, J4, and female were collected from infected roots around 2 and 6 weeks after infection using the following protocol. The harvested roots containing root-knots were washed, chopped and treated with 7.7% cellulase and 15.4% pectinase followed by rinsing in running water and filtering through a 75 μM mesh. For every five roots, 15 mL of cellulase and 30 mL of pectinase was used during enzyme treatment. Rinsing of the roots following enzyme treatment was carried out over a filter (75 μM mesh) and nematodes retained on the surface of the filter were suspended in distilled water and handpicked using a pipette under a stereo microscope. Two hundred milligrams of samples for each stage at egg, J2, J3, J4, and female were collected from the roots of tomato plants and were snap frozen using liquid nitrogen followed by storage at −80°C until further processing.

### Total RNA extraction, library preparation, and RNA-seq

To obtain high-throughput transcriptome data of *M. incognita*, we used Illumina-based NGS sequencing. RNA was extracted from nematodes collected at five developmental stages, i.e., egg, J2, J3, J4, and Fe (female) with three technical replicates at each stage. Total RNA from the samples was quantified using Nanodrop spectrophotometer (ThermoFisher Scientific), quality-assessed by RNA 6000 Nano assay kit (Agilent) and Bioanalyser2100 (Agilent). NGS sequencing libraries were generated from one microgram of total RNA using TruSeq RNA Sample Prep Kit (Illumina) according to the manufacturer's protocol. In brief, the poly-A containing RNA molecules were purified using poly-T oligo attached magnetic beads. After purification, the total poly (A)^+^ RNA was fragmented into small pieces using divalent cations under elevated temperature. The cleaved mRNA fragments were reverse transcribed into first strand cDNA using random primers. Short fragments were purified with a QiaQuick PCR extraction kit and resolved with elution buffer for end reparation and addition of poly (A). Following this, the short fragments were connected with sequencing adapters. Each library was separated by adjoining distinct multiplex identifier (MID) tag. The resulting cDNA libraries were then paired-end sequenced (2 × 101 bp) with Illumina HiSeq™ 2500 system.

### Preprocess, *de novo* assembly and mapping

Complete paired-end sequences were obtained as individual fastq files (forward and reverse) from the images by CASAVA v.1.8.2 base calling software with ASCII Q-score (offset 33). Adaptor sequences, minimum length (90 bp), bad fraction (>10%) and low quality bases with PHRED scores (Q) ≤ 20, were removed using CLC genome cell (v.4.0).

We cleaned paired-end reads using prinseq-vlite v0.19.3 with parameters (-min_len 50 -min_qual_score 10 -min_qual_mean 20 -derep 14 -trim_qual_left 20 -trim_qual_right 20; Schmieder and Edwards, 2011). *De novo* transcriptome assembly was carried out using the clean paired-end reads with the Trinity assembly pipeline (Trinity Release v2.0.2) using default parameters (Grabherr et al., 2011). The final outputs from TRINITY assembly were clustered by cd-hit-est as a set of FASTA sequences (contigs) for each stage of the nematode (Fu et al., 2012). The contigs were mapped against the reference of *M. incognita* genome (ASM18041v1) using Burrows-Wheeler Aligner (BWA) (Li and Durbin, 2009). This was followed by data summarization with bamtools stats option and python script. The summary of the transcriptomes of various stages of *M*. *incognita* are listed in Table 1. The assembled transcriptomes can be used for applications such as gene discovery or comparison of *M*. *incognita* transcriptome with other plant-parasitic nematodes to understand the molecular origin of their parasitism.

## Author contributions

B-SH and IC conceived and designed the experiments; IC, PS, DS, B-JO, and B-SH performed the experiments; IC, PS, DS, and B-SH analyzed the data; B-SH, DS, and IC contributed reagents/materials/analysis tools; PS and B-SH wrote the paper.

### Conflict of interest statement

The authors declare that the research was conducted in the absence of any commercial or financial relationships that could be construed as a potential conflict of interest.
